# A Noteworthy Case of Bilateral Idiopathic Optic Perineuritis With No Perception to Light Eye

**DOI:** 10.7759/cureus.28651

**Published:** 2022-08-31

**Authors:** Tinesh Thamotaran, Qi Zhe Ngoo, Wan-Hazabbah Wan Hitam, Azhany Yaakub, Yi Ni Koh

**Affiliations:** 1 Department of Ophthalmology and Visual Science, School of Medical Sciences, Universiti Sains Malaysia, Kubang Kerian, MYS; 2 Ophthalmology Department, Hospital Raja Permaisuri Bainun, Ipoh, MYS

**Keywords:** intravenous methylprednisolone pulse, optic perineuritis, no perception to light, schizophrenia, enhancement optic nerve sheath

## Abstract

The aim of this study is to report an interesting case of bilateral idiopathic optic perineuritis (OPN) presented with severe visual loss.

A 64-year-old male with underlying hypertension, hypercholesterolemia, and schizophrenia presented with consecutively sudden onset of the right eye (RE) painless blurring of vision for two weeks and left eye (LE) painless blurring of vision for three days. The patient has no other symptoms such as red-eye, floaters, or headache. The patient had constitutional symptoms of loss of weight for the past three months; otherwise, he has no loss of appetite or persistent low-grade fever. Upon examination, RE visual acuity was no perception to light (NPL) at all quadrants, LE 6/36, and not improved with pinhole. The relative afferent pupillary defect (RAPD) was positive over the RE. Optic nerve functions of the RE were absent; meanwhile, over LE was reduced. The anterior segment was unremarkable, with no evidence of uveitis or dense cataract. Fundus examination showed diffuse 360-degree optic disk swelling with peripapillary splinter hemorrhage, mild tortuous vessel, and minimal vitreous hemorrhage inferiorly, with no evidence of neovascularization. The LE showed diffuse 360-degree optic disk swelling with normal macula and vessel. Magnetic resonance imaging (MRI) of the brain and orbit showed bilateral optic nerve sheath (ONS) enhancement with doughnut sign and tram-track sign. The patient was treated with bilateral OPN and started on intravenous methylprednisolone 1 g OD for five days followed by oral prednisolone 1 mg/kg OD with a tapering dose for one month. Patient visual acuity regained to RE 6/18 but did not improve with pinhole and LE 6/9 with full recovery of optic nerve function.

Bilateral idiopathic OPN is a rare idiopathic inflammatory condition of ONS that typically presents with recurrent painless loss of vision with good recovery outcomes with intravenous steroids.

## Introduction

Optic perineuritis (OPN) is a part of the main group of orbital inflammatory disorders that primarily involve optic nerve sheaths (ONS). This term was first coined by Edmunds and Lawford in 1883 as histopathological infiltration of inflammatory cells around the optic nerve with preserved optic nerve function [1]. OPN can be a presenting feature of particular inflammatory disorders such as sarcoidosis, Wegener granulomatosis, or giant cell arteritis and infectious causes such as tuberculosis or syphilis. However, in the majority of cases, it is idiopathic. This entity is often missed or mistakenly diagnosed as optic neuritis (ON) due to its similarities leading to frequent relapses and inappropriate treatment. Common presenting features of OPN commonly are unilateral loss of vision associated with painful eye movement. Combination features of clinical findings and radiological features help in differentiating these entities.

In this article, we reported an interesting feature of OPN presented with bilateral OPN with severe vision loss and almost complete recovery after prompt initiation of systemic corticosteroids.

## Case presentation

A 64-year-old male with underlying hypertension, hypercholesterolemia, and schizophrenia presented with consecutively sudden onset of the right eye (RE) painless blurring of vision for two weeks and left eye (LE) painless blurring of vision for three days. The patient has no other symptoms such as trauma, red-eye, floaters, or headache. The patient had constitutional symptoms of weight loss for the past three months; otherwise, he has no loss of appetite or no persistent low-grade fever. There was no recent immunization or exposure to any toxic toxins or recent contact with active tuberculosis patients. The patient also denied any supplement, traditional medicine intake prior to symptoms, and high-risk behaviors.

**Figure 1:**
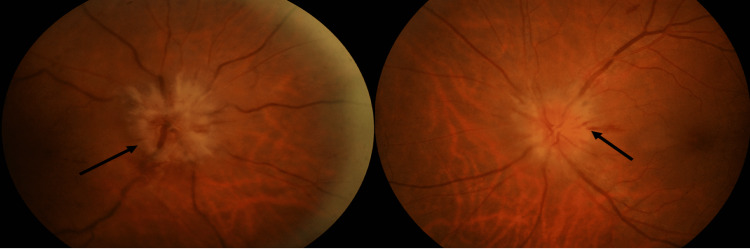
Fundus photo upon presentation. Note that (black arrow) marked optic disk swelling with splinter hemorrhages surrounding the optic disk.

**Figure 2:**
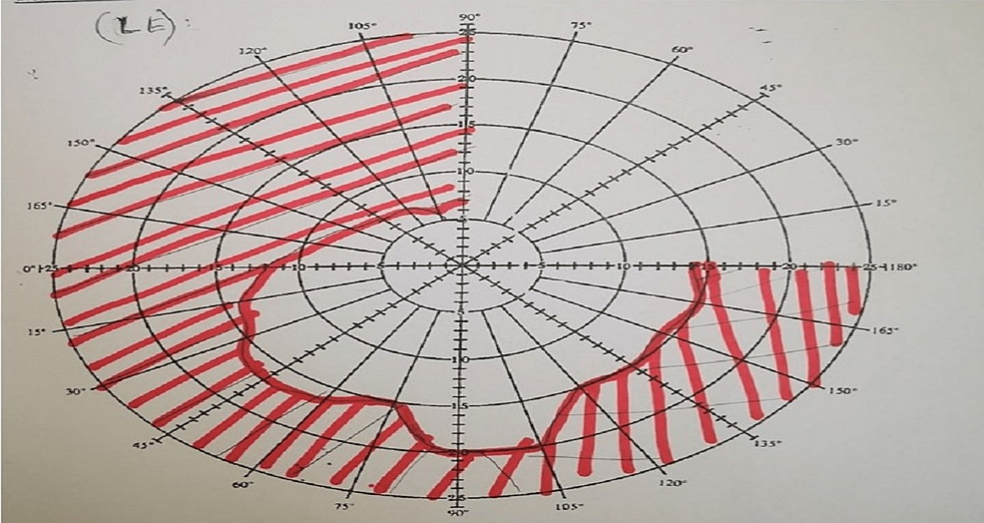
Bjerrum at presentation. Unable to perform over the RE due to poor vision (NPL). Note that 270-degree peripheral visual constriction spares the central vision.

Upon examination, the RE visual acuity was non-perception to light at all quadrants, LE 6/36, and no improvement with the pinhole. RAPD was positive in the RE with normal pupillary reaction over the LE. Optic nerve functions such as light brightness, red desaturation, and color vision over the RE were absent, whereas they were reduced by 50% over the LE. The LE visual acuity deteriorated two days after his initial presentation to no perception to light (NPL). The anterior segment was unremarkable with no evidence of uveitis or dense cataract. RE fundus examination showed diffuse 360-degree optic disk swelling with peripapillary splinter hemorrhage, mild tortuous vessel, and minimal vitreous hemorrhage inferiorly with no evidence of neovascularization (Figures 1, 2). The LE showed diffuse 360-degree optic disk swelling with normal macula and vessel (Figures 1, 2). Apart from impaired optic nerve function, the rest of the neurological assessments were normal with negative Lhermitte's signs. Systemic examination revealed a prominent superficial temporal artery; however, it was not tender upon palpation. Differential diagnoses on board this particular time raised intracranial pressure, ischemic optic neuropathy, paraneoplastic syndrome, and bilateral ON.

Baseline blood investigations such as full blood count, renal profile and liver profile, blood pressure, and random blood sugar were normal. Infective markers for syphilis, hepatitis B and C, human Immunodeficiency virus (HIV), and Mantoux were normal. Inflammatory and tumor markers such as erythrocyte sedimentation rate, C-reactive protein, antinuclear antibody, complements 3 and 4, prostatic specific antigen, carcinoembryonic antigen, alpha-fetoprotein, carbohydrate antigen 19.9, and lactate dehydrogenase were normal. The chest x-ray was normal with no evidence of perihilar or pulmonary opacity suggestive of tuberculosis, sarcoidosis, or Wegener’s granulomatosis.

**Figure 3:**
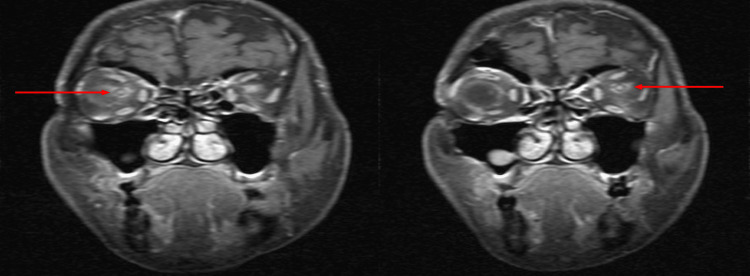
MRI orbits: the orbit T1-weighted fat-suppressed coronal view. Note that red arrows show the classical “doughnut sign” representing optic nerve sheath enhancement.

**Figure 4:**
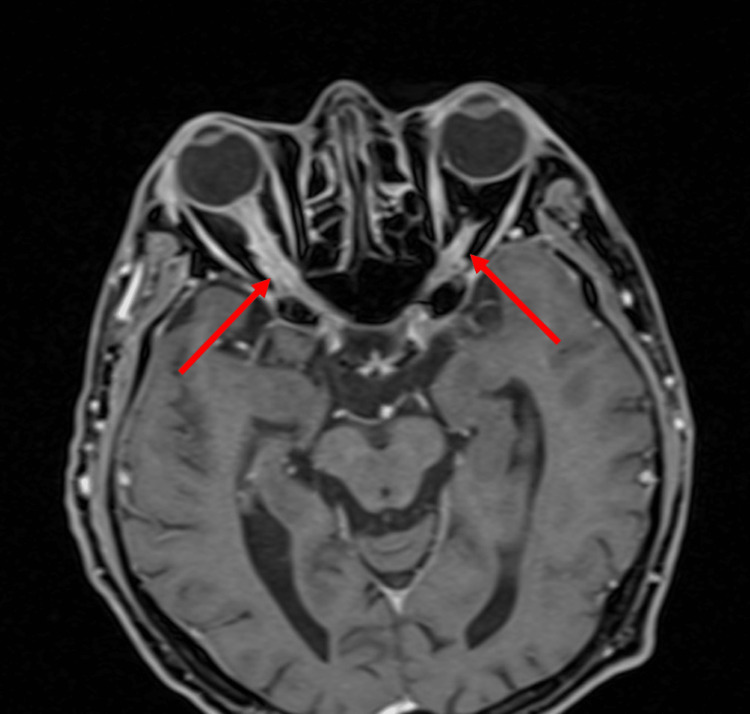
MRI brain T1-weighted VIBE fat-suppressed axial view. Red arrows show optic nerve enhancement involving the entire length of the intra-orbital optic nerve. The classical "tram-track” sign can be appreciated here. VIBE: Volumetric interpolated breath-hold examination.

Magnetic resonance imaging (MRI) of the brain and orbit showed bilateral ONS enhancement (Figures 3, 4).

**Figure 5:**
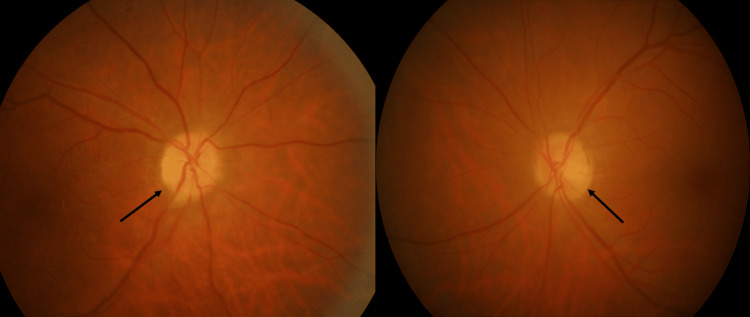
After one-month post-treatment with an oral corticosteroid, the bilateral eye optic disk swelling subsided (black arrows).

**Figure 6:**
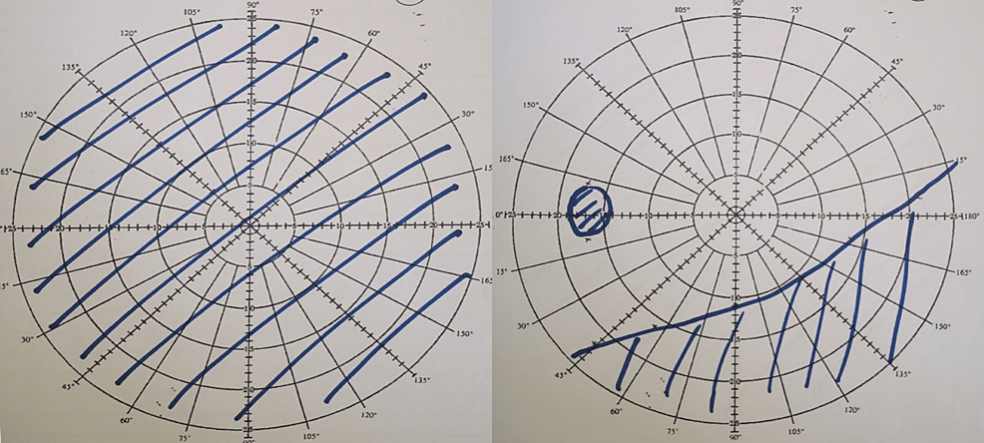
Repeat Bjerrum after initiation of pulsed IV methylprednisolone for three days. Note the RE total scotoma that tallies with persistent visual acuity of NPL and improved peripheral scotoma over LE with residual at the inferior quadrant. The blind spot of LE is not enlarged. NPL: No perception to light; LE: Left eye; RE: Right eye.

**Figure 7:**
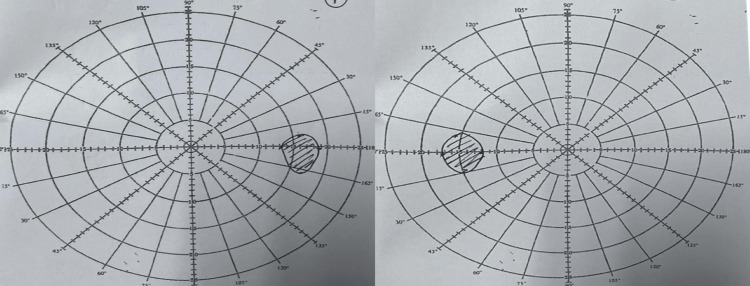
Bjerrum after one month of oral corticosteroid treatment and complete recovery of bilateral eye scotoma.

The patient was treated as bilateral OPN and started on intravenous methylprednisolone 1 g OD for five days followed by oral prednisolone 1 mg/kg OD with a tapering dose for one month and kept at 10 mg OD for three months before being stopped. The patient regained visual acuity to 6/18 RE with no improvement with the pinhole. The LE vision improved to 6/9 with full recovery of optic nerve function (Figures 5-7). The patient is currently under regular follow-up with no recurrence since then.

## Discussion

Optic perineuritis (OPN) was first coined by Edmunds and Lawford in 1883 [1]. They divided it into two groups that are exudative and purulent forms. The exudative form has a localized non-purulent pachymeningitis, whereas the purulent form is described as pachymeningitis that has involved up to the subarachnoid layer of the optic nerve. Both these forms are said to have abnormal optic nerve function except for an enlarged blind spot. OPN can be often mistaken for optic neuritis (ON) leading to inappropriate treatment and leads to frequent relapses. Histologically, the OPN hallmark feature is chronic granulomatous inflammation [2]. Few studies have done a full-thickness biopsy review of OPN showing polymorphous lymphoid infiltrate consisting of lymphocytes, plasma cells, macrophages, and polymorphonuclear cells [3,4]. Meanwhile, ONS thickening due to chronic inflammation such as infiltration and fibrosis is also seen [1]. Vasculitis changes may be seen [4]. Despite the inflammation surrounding the perineural area, vision loss is mainly attributed to the secondary infarction of the optic nerve due to either direct compression of the optic nerve or ischemia due to vasculitis [1,3,4]. More recently, OPN has been classified as a subset of a large group of the orbital inflammatory disorder called idiopathic orbital inflammatory diseases (IOID), which is also known as orbital pseudotumor [5]. They have a variety of clinical manifestations from focal to diffuse involvement of orbital structures such as myositis, dacryoadenitis, scleritis, or perineuritis. Most of these cases are idiopathic; however, associations with inflammatory disorders such as sarcoidosis, giant cell arteritis, syphilis, and Wegener’s granulomatosis were also documented [1,5-7].

Clinical presentation

OPN usually presents with acute or subacute monocular visual loss, with eye pain exacerbated by ocular movement [1]. Clinically, OPN may present almost similar to acute demyelinating ON with presenting features of acute vision loss associated with eye pain upon movement and either normal optic disk or swollen optic disk. Bilateral eye involvement is extremely rare [1,8,9]. There are a few features that might help clinicians to differentiate the entities [8,10]. Unlike ON, the OPN age group is much broader. Purvin et al. showed that the age range for OPN is 24-60 years (mean 41) contrary to ON where the range is 15-45 years (mean 35) [1]. A more recent study of case series from 2016 reported a mean age of 57 (range 15-85) in patients with idiopathic OPN [11]. Presentation of symptoms might be delayed as the onset of the disease is subacute and progresses slowly, and is described as dimming or foggy either generalized or multiple spots in nature. The visual acuity abnormalities can be as good as 6/12 and can be worser than 6/60. In our case, the visual loss is severe where NPL was detected at all quadrants over the RE and 3/60 over the LE. Typically, OPN will have associated features of dull aching pain at the retro-orbital or the eye itself, which will aggravate upon movement of the eye or palpation of the eye [10]. Usually, OPN is accompanied by mild photophobia and occasionally diplopia, ptosis, conjunctiva injection, or chemosis [1].

Visual field defects are usually present in almost all cases. Even though OPN pathology mainly involves perineural sheath, due to secondary insult to the optic nerve, visual field defects can be seen. The visual field defect mostly consists of para-central or central scotomas followed by arcuate scotomas, peripheral constriction, and altitudinal loss. Although the visual field defect is variable, OPN tends to spare central vision [1]. In our case, the patient’s RE was unable to elicit visual field defect due to severe visual acuity loss and inferior field defect in the LE. Disk swelling present in half of the reported cases, with hyperemia and splinter hemorrhages, were also present. The remaining half had either normal optic disks or pale optic disks [1].

Radiological features

Differential diagnoses of bilateral OPN include space-occupying lesions with raised intracranial pressure and ON. MRI plays a vital role in differentiating between these disorders and OPN. Typical MRI features of OPN are the “tram-track” sign of the ONS on axial views and the “doughnut sign” on coronal views [1]. These features are best seen in fat suppression and post-gadolinium orbital views, compared to a plain or contrast-enhanced CT scan [1]. Infrequently, thickening of the ONS or optic nerve can be seen. Rarely, enhancement of periorbita such as extraocular muscles, sclera, or the optic nerve itself may occur, presumably due to the inflammation of intraneural pial septa and ONS. In some cases, streaky enhancement of orbital fat can be seen, and this feature is absent in typical demyelinating ON [1]. MRI brain typically finds no abnormal signal intensities or enhancement that helps to differentiate with demyelinating ON.

Treatment

Distinguishing OPN from ON prior to initiating treatment is vital as it carries different treatment plans and possible outcomes. Patient with ON has a higher risk of developing multiple sclerosis (MS), while OPN has no predisposed risk toward MS. Second, corticosteroid treatment has not improved the visual outcome in ON where it merely only fastens the recovery process, and expectant management also has shown recovery of vision [12]. On contrary, OPN benefits greatly from corticosteroids in terms of visual recovery and usually responds greatly. Progressive vision loss will occur in OPN patients if the initiation of corticosteroid treatment is delayed [1]. The outcome of OPN is dependent on the timing between the onset of symptoms and initiation of corticosteroid treatment [1].

Administration of corticosteroids also slightly differs from ON. Optic Neuritis Treatment Trial (ONTT) showed that administrating oral corticosteroid alone increases recurrent rate, not in OPN [1,13]. Moreover, in ON, oral steroids are given for 11 days following high-dose intravenous steroids for three days. However, in OPN, oral steroids are given for a prolonged duration to reduce the risk of relapse and indirectly reduce the repetitive damage to the optic nerve. Hence, we can deduce that systemic, either oral or intravenous, steroids for prolonged duration have proven to be beneficial for OPN in terms of visual recovery and prevention of recurrence. Purvin et al. recommended a regimen of 80 mg OD for OPN. The case series mostly were treated with oral prednisone, either 60 or 80 mg daily, and tapered over a few weeks to months. All the patients benefited in terms of improvement in ocular pain and vision.

Relapse occurred in four cases upon tapering steroid dosage, for which supplementary treatment was given (intravenous and peribulbar steroids, immunosuppressant, and radiation therapy) [1]. Another case report by Ohtsuka et al. imitated the ONTT trial by administrating intravenous methylprednisolone 1 g for three days followed by 30 mg oral prednisolone for 14 days. The treatment regimen showed rapid recovery in visual acuity, optic disk swelling, and complete recovery of abnormal enhancement of optic nerve in MRI. Similar pulse steroid therapy was also mentioned in several studies that showed positive outcomes with no recurrence [14].

Our patient was given intravenous methylprednisolone 250 mg QID for three days and was followed by oral prednisolone 1 mg/kg OD with a tapering dose for one month and kept at 10 mg OD for three months before being withheld. Fortunately, our patient has not suffered from recurrence ever since the first episode and is on regular follow-up without oral corticosteroid. We believe that this case is particularly unique because the bilateral incidence of idiopathic OPN in one setting is rare.

## Conclusions

In conclusion, OPN is a subset of IOID where its main insult area involves the ONS and its surrounding tissue. Its clinical features are almost similar to ON; however, certain features and radiological findings can help distinguish these two. Various etiology are reported to cause OPN, and extensive blood investigations are required. MRI imaging is proven to be beneficial in the diagnosis and differentiation of ON. Treatment should be tailored according to the cause, particularly idiopathic OPN; prompt and prolonged high-dose corticosteroid is warranted in order to prevent irreversible visual loss.
